# Advancing preference testing in humans and animals

**DOI:** 10.3758/s13428-025-02668-5

**Published:** 2025-06-06

**Authors:** Dana Pfefferle, Steven R. Talbot, Pia Kahnau, Lauren C. Cassidy, Ralf R. Brockhausen, Anne Jaap, Veronika Deikun, Pinar Yurt, Alexander Gail, Stefan Treue, Lars Lewejohann

**Affiliations:** 1 Welfare and Cognition Group, Cognitive Neuroscience Laboratory, German Primate Center–Leibniz Institute for Primate Research, Kellnerweg 4, 37077 Göttingen, Germany; 2 Leibniz-Science Campus Primate Cognition, German Primate Center & University of Göttingen, Göttingen, Germany; 3 Institute for Laboratory Animal Science, Hannover Medical School, Hannover, Germany; 4 German Federal Institute for Risk Assessment (BfR), German Centre for the Protection of Laboratory Animals (Bf3R), Berlin, Germany; 5 Population and Behavioral Health Services, California National Primate Research Center, University of California, Davis, CA USA; 6 Institute of Animal Welfare, Animal Behavior and Laboratory Animal Science, Freie Universität Berlin, Berlin, Germany; 7 Department of Sociobiology/Anthropology, Johann-Friedrich-Blumenbach Institute for Zoology, Georg-August University, Göttingen, Germany; 8 Georg-August University School of Science, Göttingen, Germany; 9 Bernstein Center for Computational Neuroscience, Göttingen, Germany

**Keywords:** Preference, Rating, Ranking, Scaling, Binary choice, Welfare, Rhesus macaque, Mice, Human

## Abstract

Preference tests help to determine how highly individuals value different options to choose from. During preference testing, two or more options are presented simultaneously, and options are ranked based on the choices made. Presented options, however, influence each other, where the amount of influence increases with the number of options. Multiple binary choice tests can reduce this degree of influence, but conventional analysis methods do not reveal the relative strengths of preference, i.e., the preference difference between options. Here, we demonstrate that multiple binary comparisons can be used not only to rank but also to scale preferences among many options (i.e., their worth value). We analyzed human image preference data with known valence scores to develop and validate our approach to determine how known valence ranges (high vs. low) converge on a scaled representation of preference data. Our approach allowed us to assess the valence of ranked options in mice and rhesus macaques. By conducting simulations, we developed an approach to incorporate additional option choices into existing rank orders without the need to conduct binary choice tests with all original options, thus reducing the number of animal experiments needed. Two quality measures, consensus error and intransitivity ratio, allow for assessing the achieved confidence of the scaled ranking and better tailoring of measurements required to improve it further. The software is available as an R package (“simsalRbim”). Our approach optimizes preference testing, e.g., in welfare assessment, and allows us to efficiently and quantitatively assess the relative value of options presented to animals.

**Supplementary Information:**

The online version contains supplementary material available at 10.3758/s13428-025-02668-5.

## Introduction

Preference tests are a straightforward approach for investigating how different options are appraised. However, if one option is known to be preferred over another, the magnitude of this preference is still unknown. Therefore, methods that not only allow rank to be estimated (i.e., the order in which the options are preferred) but also scale preference strength (i.e., how much option A is valued over option B) are necessary to gain a better understanding of the valence of the choices that are made.

While humans can be asked directly how items rank and about the strength of these preferences (“scaled ranking”), determining the preferences of animals is much more challenging and usually derived from indirect methods (e.g., amount consumed). In principle, more than two options can be evaluated against each other in a single test, although the maximum number of testable options is limited (Habedank et al., 2018). To overcome such limitations, we propose a method that combines the outcome of multiple binary choice tests.

### Binary choices versus multiple choices

Simultaneously presented options always influence each other to some extent. For example, eating chili peppers raises the preference for milk afterward because it helps handle capsaicin, the oily chemical compound responsible for the burning sensation (Nolden et al., 2019). Moreover, options presented in the same trial might be perceived as belonging together, as one condition, instead of as two separate entities to choose from (especially relevant in home cage-based choice tests, see Hobbiesiefken et al., 2021; Lewejohann & Sachser, 2000; Patterson-Kane et al., 2001). Presenting multiple options simultaneously (e.g., bedding material in mice: Blom et al., 1992; Godbey et al., 2011; Kawakami et al., 2007, 2012) instead of binary comparisons (e.g., bedding material: Baumans et al., 2002; Freymann et al., 2015; Kirchner et al., 2012) might, however, lead to even more cross-influences. For example, Tordoff (2003) showed that the overall amount of liquid consumed by mice increased as the number of bottles to choose from increased. In addition, the number of trials must increase with an increase in options to detect statistically significant differences (Raffa et al., 2002). Therefore, conducting repeated pairwise comparisons might provide more reliable results than presenting all possible options simultaneously in one trial. However, repeated pairwise comparisons can be time-consuming (Hobbiesiefken et al., 2023).

### Testing preferences in animal studies

Typically, preference tests are designed so subjects can choose between two options. The option chosen (measured by, e.g., shortest latency to approach, longer inspection/manipulation duration, and the amount consumed) is then said to be preferred. On the other hand, less preferred options either are not chosen, or animals will work to avoid them (Kahnau et al., 2023; Rushen, 1986). For example, a binary choice between two branches of a corridor is offered in a T-maze-based preference test design, where different options are placed at the end of the branches. The option presented in the branch entered first or the branch in which more time was spent is considered to be preferred over the option offered in the opposing branch (Cutuli et al., 2015; Dawkins, 1977; Leenaars et al., 2019; Ras et al., 2002; van der Plasse et al., 2007). However, it is unclear how strong a measured preference is when comparing only two options. A lack of preference, moreover, may be due either to the animal rating both options equally or to not “answering” the question for other reasons (Kirkden & Pajor, 2006). Such a lack of clarity was the case for a T-maze preference test in mice choosing between almond milk and apple juice (Habedank et al., 2021). Although previous tests have indicated that these mice strongly preferred almond milk, they alternated between the two T-maze branches equally overall and rarely consumed any of the liquids offered. The authors concluded that, in that study, the T-maze design was unsuitable for testing the liquid preferences of mice. Thus, identifying a suitable preference test design is an important step before conducting successive binary choice tests to rate multiple options (Hobbiesiefken et al., 2023).

### Transitivity

The combination of multiple bimodal choices can be used to derive a ranking of multiple options. Typically, the combination is based on the assumption of transitivity: if one option, A, is preferred over another, B, and B is preferred over option C, then A should be preferred when directly compared to C (Pastor-Bernier et al., 2017). However, intransitive triplets may occur, for example, if different motivations are mixed within the same preference test or if underlying values (deliberately) form a non-transitive hierarchy (e.g., rock-paper-scissors-lizard-spock [Lorre, Chuck. “The Big Bang Theory Video.” Retrieved June 26, 2024: www.youtube.com/watch?v=pIpmITBocfM]). Although analyses of multiple human and animal preference data showed that violations against transitivity are relatively scarce (Regenwetter et al., 2011), the derived linear order from multiple binary comparisons of options should be evaluated against the occurrence of such intransitivity. To reflect this, we propose calculating the “intransitivity ratio” as one quality measure of the derived ranking.

### Combining binary choices

It is reasonable to assume that a meaningfully ordered sequence can be obtained by testing several options in pairs against each other in all possible combinations. Such a sequence allows us to derive the ordinal strengths of the preferences, with options at the top of the scale being strongly preferred over options in the middle or at the bottom. The distances between all items would be the same in a perfect linear system. This assumption, however, is not reasonable for options occurring in real life, where the distances are most likely spaced unequally across the scale. Therefore, calculating the individual positions on an ordered scale rather than merely calculating a rank order provides additional information about the preference system (Hatzinger & Dittrich, 2012). The proposed “worth values” (Hatzinger & Dittrich, 2012) represent probabilities for each option chosen in binary comparisons normed to a scale between 0 and 1. On a general note, the number of options used in such an approach will affect how easily options can be separated, with more options leading to less confident overall scaling. This effect is due to the overall sum of worth values adding up to 1, so the mean difference between options decreases as the number increases.

### Aggregation of individual preferences

A statistical sample of many individual preferences is drawn to derive an objective, generalizable ranking that allows statements to be made about the preferences of a studied population. In other words, several individuals are presented with a series of binary choices, while the scaling is aggregated for the entire group. However, personal preference does not necessarily reflect group preference (Hobbiesiefken et al., 2023), which can result in intransitive overall rankings. Moreover, aggregating discrepant individual choices may lead to overall rankings not reflecting the population’s perspective. To address this issue, we propose the “consensus error,” a measure of confidence in aggregated scaling concerning individual choices.

### Simulations

Once a scaled ranking of several options is known, incorporating additional options may be desired. Due to time and resource limitations, conducting all possible binary choice tests for extending a scaled ranking might not be feasible. The number of possible combinations for *n* options can be calculated by (*n* choose 2) = n*(*n* – 1)/2. Therefore, the number of possible combinations increases considerably for each new option added. Here, we propose a simulation approach that we tested on empirical preference test data. Based on our results, we suggest a workflow for generating scaled ranks of items from incomplete binary comparisons. In addition, our simulations allow us to estimate how ties between options (i.e., an unknown preference relationship between two options) influence the scaled ranking. Dealing with ties becomes especially interesting in cases where the threshold for preference is set at more than 50% confidence, i.e., to ensure a reasonably strong preference for one good over another. However, increasing the threshold above 50% automatically leads to an increased chance of ties. For example, when a preference is only considered valid if an option was chosen over 65% of the time, all ratios between options falling within 35:65 and 65:35 must be viewed as a tie. On the contrary, a threshold of 50% would only have a slight chance of being counted as a tie, namely, if the ratio were precisely 50:50.

It is worth noting that simulations can significantly reduce the number of experiments required. This quantitative method saves time and improves animal welfare by reducing the frequency of animal experiments (3Rs: reduce).

To predict the accuracy of simulated data, we propose two quality measures (i.e., consensus error and intransitivity ratio). For details on calculating these quality measures, see the methods sections “Transitivity” and “Consensus error.”

### Purpose of the present study

In our study, we used multiple binary comparisons to demonstrate that a combined analysis of binary choice tests can be used to derive an overall scaled ranking of tested options for different species. First, we analyzed human preference data for images with known valence scores in sets that ranged either low or high in overall valence. The derived knowledge of how known valences converge on a worth-value plot can be used to estimate the valence of ranked options in animal experiments. Second, we included preference data from mice (*Mus musculus*) and rhesus macaques (*Macaca mulatta*) to exemplify the applicability of our approach for animal experiments. Both species of animals could choose between various liquids presented in all possible binary combinations.

The calculated worth values reflect the probability of each option being preferred. To evaluate the quality of the derived scaled ranking, we introduce two quality measures: intransitivity ratio and consensus error. We hypothesize that the proposed quality measures will be related to the range of valence and thus provide indirect information on the valence of the tested options. Rankings comprising elements low in their valence range (i.e., on average being perceived very similar) are more likely to yield higher consensus errors and a larger intransitivity ratio (i.e., more disagreement between subjects, hence a larger number of overall intransitive choices). This does not exclude the fact that on the individual level, e.g., due to different experience, surprising discrepancies in preference order and/or strength from the population mean may occur. In addition, we evaluate how different thresholds set for defining a biologically relevant preference can affect the quality of a derived ranking.

In several simulations, we aim to demonstrate that ranking and scaling by binary comparisons is still possible with reasonable precision despite missing values or ties. We explore the feasibility of integrating new options into scaled rankings by conducting only a fraction of the possible binary comparisons. In cases where the desired precision of a scaled ranking can be set in advance, one could estimate a realistic number of binary comparisons that would have to be conducted. This way, a simulation approach can reduce the number of necessary comparisons (i.e., animal experiments) to integrate a new option into an existing ranking.

## Methods

### Humans

For preference rating, two sets of seven pictures each (set depicting images with a low valence range: sunset, flowers, food, grass, house, monkey, timber; set depicting images with a high valence range: lake, cat, crow, doctor, fire, human posture–frustrated, war; see supplementary Table S1) were taken from the OASIS database (Kurdi et al., 2017) and presented to three groups of subjects. The high-valence set was rated by 25 participants (11 male, 14 female) with an average age of 25.6 years (range: 19–87). The second set (low valence) of pictures was rated by 48 participants (24 male, 23 female, 1 other) with an average age of 24.7 years (range: 19–40). The participants were recruited at the University of Göttingen, Germany, and the test was conducted in 2017 in a controlled laboratory environment. The first set of pictures (high–valence set) was tested a second time in 2019 during a graduate spring school by 16 participants (mean 31.1 years; range 26–40, 12 female, 4 male).

#### Experiment

The experiment was scripted using JSPsych v5.0.3, a tool to create web browser-based experiments (De Leeuw, 2015). After a short explanatory introduction to the experiment, a series of all possible 21 choices between two images out of a pool of seven images was presented. The participants were asked, for each combination, to select the image that evoked more positive feelings. The choice was made by pressing the left or right arrow key on the computer keyboard corresponding to the screen position of the chosen image. Image order and position (left or right) were randomized throughout the experiment.

#### Test pictures

We tested two image sets taken from the OASIS database (Kurdi et al., 2017). All images in this database are rated by an average of 103.25 participants concerning valence on a seven-point Likert scale. The first image set we presented comprised seven images with average valence scores ranging from 1.8 to 6.4, reflecting a high range of valence scores (see supplementary Table S1). The second image set comprised seven different images from the same database, where the average valence scores had a smaller valence range from 3.83 to 6.07 (supplementary Table S1). The experiment is available with both datasets online at http://seqpref.phenotyping.com/.

### Rhesus macaques

Six male rhesus macaques aged 6–19 years (mean = 13.09) living in same-sex groups of 2–4 individuals at the German Primate Center participated in this study. The monkeys were housed in indoor and outdoor rooms equipped with toys, wooden structures, and natural and artificial light. The space provided exceeded all applicable German and European regulations. Indoor rooms were temperature-controlled and connected by a tunnel with rooms at ambient outdoor temperature and lighting but protected from precipitation.

Each indoor room was connected to an experimental compartment in an adjacent room via a sliding door. All animals entered this compartment regularly to be fed by care staff, take part in touch-screen tasks, or be separated from the group for veterinary inspection. Prior to preference testing, the monkeys were encouraged to enter their respective experimental compartments and separate from their social group for small fruit rewards (grape, raisin, or banana). Social group members and those animals in adjacent cages were moved out of sight to prevent the experiment from being interrupted. All monkeys were familiar with this procedure. In addition, human access to the testing area was prevented during the preference test.

Preference tests were conducted once per day around noon. A test session consisted of a two-bottle choice test. The animals were not fluid-restricted on the test days (for definitions of access to water, see Pfefferle et al., 2018). Before each preference test, the monkeys had free access to water for two hours, followed by three hours of no water access. After preference testing and with a time lag of one hour (to temporally separate fluid presentation during preference testing from the general water supply), monkeys were given free access to water for at least two hours (typically much more). In addition, the monkeys received monkey chow ad libitum supplemented with dried fruits, fresh fruits, and vegetables. The health of the monkeys was monitored daily by the animal care staff, veterinarians, and scientists, all highly experienced with these animals.

For preference testing, we simultaneously presented two liquids out of a set of five, representing an expected high valence range: banana and grape juice (each diluted 1:3 with tap water), tap water, 100 mM NaCl, and 5 mM quinine. The monkeys were familiar with the liquids to be tested before the experiment, except for quinine and NaCl. In each test session, two bottles filled with 500 ml of liquid were presented simultaneously for 15 min. The distance between the bottles was 13 cm, and the bottles were covered with a cloth before the test began so that the monkeys could not see the contents in advance. All possible pairwise combinations of liquids were tested against each other and randomized for test order and bottle position. All tests were repeated with the bottle position of the respective liquids switched, to control for potential side biases. After 15 min, the amount of liquid remaining in the bottles was measured and used to determine preference according to the thresholds we investigated (here, 50% and 65%).

### Mice

We tested 11 female C57BL/6J mice at the German Federal Institute for Risk Assessment, Berlin, Germany. Mice were purchased at the age of 21 days from Charles River, Sulzfeld. All mice had different mothers and foster mothers to ensure maximum epigenetic independence. The mice were implanted with an RFID chip (radio frequency identification, Planet ID, FDX-B transponder according to ISO 11784/85), providing a unique identification number for individual differentiation by the IntelliCage system. At the start of the first preference test (low valence range), the mice were seven months old and weighed between 25.0 and 29.0 g. At the start of the second preference test (high valence range), the mice were 14 months old and weighed between 25.5 and 37.0 g. We used female mice only to avoid potential territorial behavior male mice might have displayed (e.g., monopolization of access to the IntelliCage and, hence, the liquids), which would have influenced the results of the preference testing. Estrus was not monitored. The mice were handled via the tunnel method (the tunnel was a Plexiglas tube 17.5 cm long and 4 cm in diameter; for a video tutorial, see https://wiki.norecopa.no/index.php/Mouse_handling).

All mice lived together in a home cage-based test apparatus described in Kahnau et al. (2023). Briefly, this test apparatus comprises three units: the home cage (type IV Macrolon cage, L × W × H: 598 × 380 × 200 mm, Tecniplast, Italy), an AnimalGate (TSE-Systems, Germany), and an IntelliCage (TSE-Systems, Germany). The IntelliCage is a computer-based test system that allows one to investigate how individual mice learn, whereby a mouse is identified by the system by reading its RFID transponder (Choi, 2018; Voikar et al., 2010). Each corner of the IntelliCage contains a computerized unit with an RFID reader and two liquid dispensers. The liquid consumption was measured for each mouse using a lickometer (measuring the change in conductance upon touching a mouse's tongue) connected to each liquid dispenser. The home cage contained nesting (two papers, four cotton rolls) and bedding material (spruce/fir shavings, 2.5–4 mm, JRS Lignocel FS, Germany), two red houses (“TheMouseHouse,” Tecniplast, Italy), and four wooden bars to chew on. Food (LAS QCDiet, Rod 16, autoclavable, LASvendi, Germany) was available only in the home cage, whereas liquids were only available in the IntelliCage. Access to the IntelliCage was available to each mouse via the AnimalGate, guaranteeing that only a single mouse could conduct a preference test at a time. Thus, the influence of other mice during the preference tests was prevented. See supplementary material, Fig. S1, for a schematic drawing of the home cage-based test apparatus.

The temperature and humidity in the room containing the home cage-based test apparatus were kept at 22 °C ± 3 °C and 55% ± 15%, respectively, with a light/dark cycle of 12/12 h. The ceiling light turned on at either 8:00 AM (April–October, first/low-valence range test block) or 7:00 AM (November–March, second/high-valence range test block). Sunrise was simulated via a wake-up light (Philips HF 3510, 100–240 vac, 50–60 Hz, Philips Consumer Lifestyle B.V. Netherlands) that gradually increased light intensity 30 min before the ceiling light turned on. After 1.5 h, the wake-up light switched off. The daily visual health inspection of the mice was carried out by the experimenter or caretakers between 7:00 AM and 10:00 AM.

We tested two sets of liquids, one with a presumed low valence range (first set) and the other with a presumed high valence range (second set). Between the presentation of the first and second sets, slight adjustments were made to the test design (see supplementary Table S2) to optimize the performance and facilitate the analysis. In both sets, liquids were presented in binary combinations using the home cage-based test system.

#### Habituation phase

To ensure that all mice were familiar with the different liquids, before both blocks of preference testing, they had access to the liquids in the same corner of the IntelliCage for 24 h each. Water was offered for 24 h between each liquid presentation. During habituation for the low-valence-range preference tests (first block), the order of liquid presentation was 10 mM NaCl, 5 mM sucrose, 10 mM HCl, tap water, and lastly, 10 mM sucrose. Due to technical problems with the IntelliCage system, we repeated the presentation of 10 mM sucrose. In preparation for the second block of preference tests (high-valence range), liquids were presented one at a time in all four corners in the order of apple juice (Sachsenobst Apfelsaft klar, 1:3 dilution), 3 mM quinine hydrochloride dihydrate, 10 mM HCl, almond milk (Alnatura, Mandel Drink, 1:3 dilution), and lastly tap water. All liquids were diluted with tap water. Between the habituation and test phase, there was a two-night break with free access to water.

#### Testing phase

The two-bottle preference test was carried out in corners 1 and 4 of the IntelliCage (see supplementary Fig. S1), i.e., each corner presented one of two liquids. Liquids were presented for 24 h. All possible combinations of the liquids were tested against each other and randomized for test order and cage corner. All tests were repeated with the position of the respective liquids switched to control for potential side biases. After one binary choice test cycle was completed (2 × 24 h to accommodate for side switches), a 24-h break occurred during which tap water was offered. Tap water was available in corners 1 and 4 during the first block of preference tests and in all four corners during the second block of preference tests. Each day, the corners were cleaned with 70% ethanol. Due to technical issues with the lickometer, we repeated the presentation of apple juice versus quinine and HCl versus water in the second block of preference tests. For the first preference test, the mice were allowed to drink during predefined time windows (7:00 PM–9:00 PM, 0:00 AM–2:00 AM, and 5:00 AM–7:00 AM), while in the second preference test, no such restrictions were applied.

Preferences were determined by counting the number of corner visits with licks, where a higher number indicated a preference for the respective liquid for the binary combination presented.

### Data preparation and statistical analyses

Data transformation and all statistics were carried out with the program R v.4.0.3 (R Core Team, 2023). While the data from human preference testing were binary by design (subjects were asked to choose either the left or right image), the liquid consumption by mice and rhesus macaques was measured as discrete counts (amount consumed in milliliters for rhesus macaques, number of corner visits with licks in mice). We transformed the rhesus macaques and mice data to consecutive integers, with the liquid consumed more being considered preferred, the liquid consumed less considered non-preferred, and the remaining liquid combinations counted as ties. Transformations of liquid consumption data were performed along the lines of two exemplary preference limits, 50% and 65%. While with the 50% preference limit, a tie is set for equal preferences (ratio of 50:50), a 65% preference limit sets ties for responses that fall within the ratios of 35:65 to 65:35.

We fitted log-linear Bradley–Terry (LLBT) models to test the species'relative preferences for certain liquids (rhesus macaques and mice) or images (human), respectively. The LLBT model is implemented in the R package “prefmod” ([r-package], Hatzinger & Dittrich, 2012; Hatzinger & Maier, 2023). Running a LLBT model requires the data to be in single-line format per subject. To transform the data from more common formats of experimental data, a function ‘bimload’ of our newly compiled R package “simsalRbim” was used. Within “simsalRbim,” the respective data reformatting is carried out by the function “dcast” of the package “reshape2” (Wickham, 2007). The final design matrix needed to run the LLBT model is achieved using the function “llbt.design” embedded in the “prefmod” package (Hatzinger & Dittrich, 2012; Hatzinger & Maier, 2023). The generalized nonlinear model is fit using the function “gnm” with a Poisson error structure, providing a numerical summary of the statistical output. Based on the model estimates provided by “gnm,” the function “llbt.worth” (package “prefmod”) calculates the worth values for all presented options (here, liquids or images). The worth values of all options sum up to one, where higher numbers indicate a higher probability of preference for the respective option. The distance between the options on the worth value scale (ranging from 0 to 1) is a good proxy describing the rank order and a relative valence derived from binary comparisons (scale). To estimate the quality of the derived scaling, we introduced the intransitivity ratio and consensus error as two measures within the “simsalRbim” package.

#### Transitivity

The rankings per individual of all three species were tested for intransitive triples using the “simsalRbim” R package. To briefly explain this concept, assume there is a ranking among three options, A, B, and C. Overall, there are eight possible outcomes for ranking all sequential binary comparisons of the three options. While six of these combinations give clear rankings, two combinations would lead to intransitive triples (i.e., A > B > C > A, C > B > A > C), which would challenge a linear ranking. Aside from intransitive triples, more complex intransitive relations may also be observed: an intransitive quadruple would consist of four options A, B, C, and D which can be ordered in six different intransitive circles (A > B > C > D > A, A > B > D > C > A, A > C > B > D > A, A > C > D > B > A, A > D > B > C > A, and A > D > C > B > A). However, each intransitive quadruple contains at least one intransitive triple. (For example, within the intransitive quadruple A > B > C > D > A, the relation A to C can either be A < C or A > C. If A < C, there is an intransitive triple with A > B > C > A. If A > C, then there is an intransitive triple with A > C > D > A.) The same applies to all intransitive *n*-tuples with *n* > 3, each containing at least one intransitive triple. Thus, counting all intransitive triples within a ranking is sufficient to measure intransitivity.

While intransitivity may very well occur in real-world data from humans and animals, it has been reported to occur rarely, i.e., less than 5% of the data (Regenwetter et al., 2011). We use the overall amount of intransitivity within a ranking and calculate the intransitivity ratio as a quality measurement for the ranking (https://tinyurl.com/simsalRbimquality).

#### Consensus error

Several subjects, who do not necessarily agree with the overall ranking of the population, ranked each option with respect to all other options. The consensus error allows us to estimate subject agreement for a derived ranking. In a nutshell, the consensus error is calculated by iterating through all possible option combinations and counting the percentage of disagreement. The mean percentage of disagreement for each option was scaled to a value between 0 and 100%. A consensus error value of 0% indicates perfect agreement of a ranked position. In comparison, a consensus error of 100% indicates complete disagreement, meaning that for all binary choices made concerning option A and all other items X, half of the raters chose option A, while the other half chose option X. It must be noted that the consensus error is affected by the number of raters as well as by the number of options to be ranked. When only a few raters are included, the resulting consensus error is significantly more affected by each single rater; likewise, the fewer the options, the more the consensus error is affected by a single disagreement between two option positions (https://tinyurl.com/simsalRbimquality).

#### Correlation analyses

The OASIS image database provides values for the valence of each image based on ratings of approximately 100 participants (see supplementary material, Table S1). This additional knowledge allows us to measure the validity of the proposed method for calculating worth values from binary comparisons by relating worth values to the known valence. We consider our human worth values to be at least ordinal since they indicate a ranking. Therefore, Spearman's rank correlations (function “cor.test()”) were calculated using the valence scores and the worth values derived from the multiple binary comparisons.

### Simulations

We performed several simulations based on our data on mice to outline how to extend existing scaled rankings with additional options. For example, if there is a ranking A > B > C > D, performing all pairwise comparisons to integrate a new option X into the ranking might not be necessary. Assuming full transitivity (see above) of the given ranking, X is compared to B first; if X > B, then X must only be compared to A to receive a comprehensive rating. If X < B, then an additional comparison to D would be warranted; if X > D, another comparison of X to C would be necessary. However, the assumption of full transitivity might not always be met. Therefore, the allowed intransitivity ratio (a value between 0 = full transitivity and 1 = full intransitivity) can be included in the simulation, and all simulated preferences leading to rankings with an intransitivity ratio above the threshold are discarded. Note that in our package “simsalRbim,” the intransitivity cutoff is preset to 0.1, i.e., all values above this cutoff are discarded. Such a simulation is termed “informed.” In contrast, simulations that do not include any assumptions regarding the (in)transitivity of the final ranking are termed “uninformed” and contain all possible simulated pairwise outcomes.

We used the experimental data for our simulations as a known ground truth. We removed single options from the raw data and reintroduced them by only including comparisons to a subset of the remaining options. We could thereby simulate and verify the respective position of the (previously removed and now new) option using this partial information from the original data. In contrast, the comparisons with the remaining options were simulated. We compared the options'known “true” position with the simulated positions by calculating the frequency of absolute counts. The frequency of absolute counts is also expressed as the frequency of true positives standardized to the number of simulation rounds. The simulations were conducted either uninformed (including all simulated outcomes) or informed (optimizing simulated outcomes to minimize intransitivity).

Scaling based on worth values was also used for the simulations, above the mere ranking of the data. In the simulation, the worth function uses binary comparisons of all options to achieve a worth ranking, which is repeated *n* times for all possible combinations. In this step, the relations of the simulated option positions are randomized, resulting in *n* worth comparison sets. The precision of worth values increases with the number of worth comparison sets. We used 95% confidence intervals as precision estimates for the options'relative position within the scaled ranking. The consensus error was used as a quality measure to validate the scaled rankings.

## Results

### Humans

**Fig. 1:**
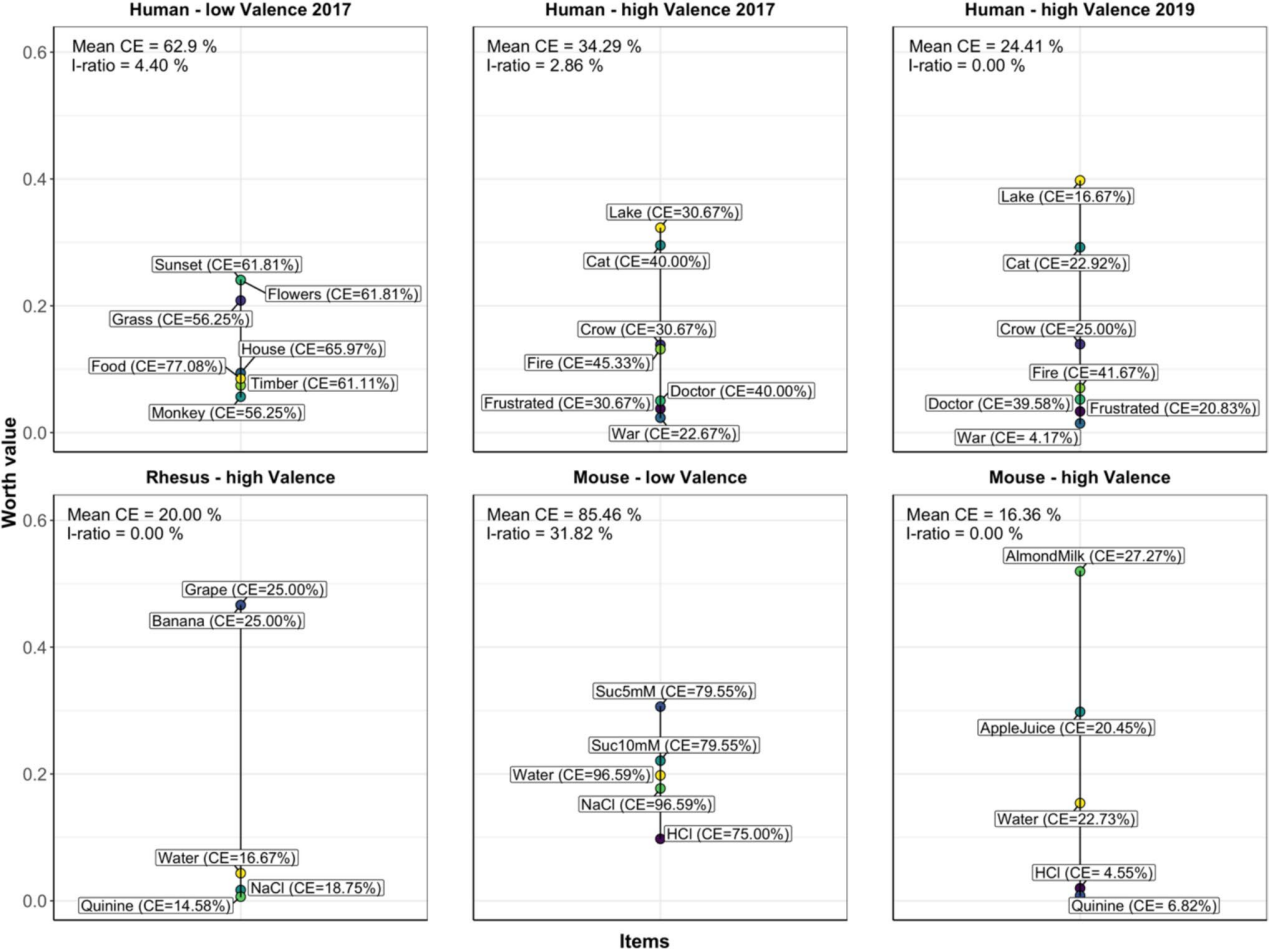
Preference ranking of picture sets presented to humans and liquids presented to mice and rhesus macaques. The preference threshold was set to 50%. The consensus error (CE; higher values indicate more disagreement between subjects) and the intransitivity ratio (I-ratio; higher values indicate more intransitive choices) are given as quality measures. *Note.* The percent transitivity can be derived as 100 – (I-ratio)

Human subjects were presented with two sets of images that differed in the range of their valence. In the set of pictures with a high valence range, a relatively distinct rank order (lake, cat, crow, fire, doctor, frustrated, war) was determined (study from 2017) and replicated in a second study conducted two years later (2019). In the first study of participants, the overall consensus error was at 34.29%, indicating that about 16 out of 25 agreed with the same order of ranking (Fig. 1). There were only a few intransitive choices made, leading to an intransitivity ratio of 2.8%. The consensus error in the second study was lower (24.41%), and no intransitive choices were made. In both runs, the lowest consensus error value for individual options was for the picture of a boy holding a gun (war), indicating that the overall agreement in the low ranking of this picture was the greatest.

The picture set representing a low valence range revealed an overall consensus error of 62.9%, indicating little consensus among the participants, i.e., 30 out of 48 participants disagreed with the order of their ranking. Likewise, the intransitivity ratio was higher (4.4%) for this picture set than that of the high-valence picture set.

We performed Spearman's rank correlation analyses using the derived worth values and the known valence scores from the OASIS database to evaluate the scoring from multiple binary comparisons. For the high-valence picture set tested in 2017, a significant positive correlation (*ρ*(5) = 0.96, *p* = 0.003) was found. Presenting the same picture set to different participants in 2019 revealed a similar positive correlation (*ρ*(5) = 0.96, *p* = 0.003). A significant positive correlation was also found for the low-valence picture set (*ρ*(5) = 0.86, *p* = 0.024), although the amount of variance explained and the *ρ*-value is lower. Overall, the correlations indicate that the method for ranking binary choices reveals results comparable to valence scorings obtained from the OASIS database.

In the human data, all choices were set to the threshold level of 50%, i.e., participants were given the choice between one or the other picture. Hence, results for different thresholds are not available for this dataset.

### Rhesus macaques

Five liquids from a prospectively high valence range were tested in rhesus macaques. Overall, the sweet-tasting liquids grape and banana were preferred equally over tap water. Bitter-tasting quinine solution and salty-tasting NaCl were mostly avoided. The overall consensus error was low, at 20%, and there were no intransitive choices on an individual level (Fig. 1).

**Fig. 2:**
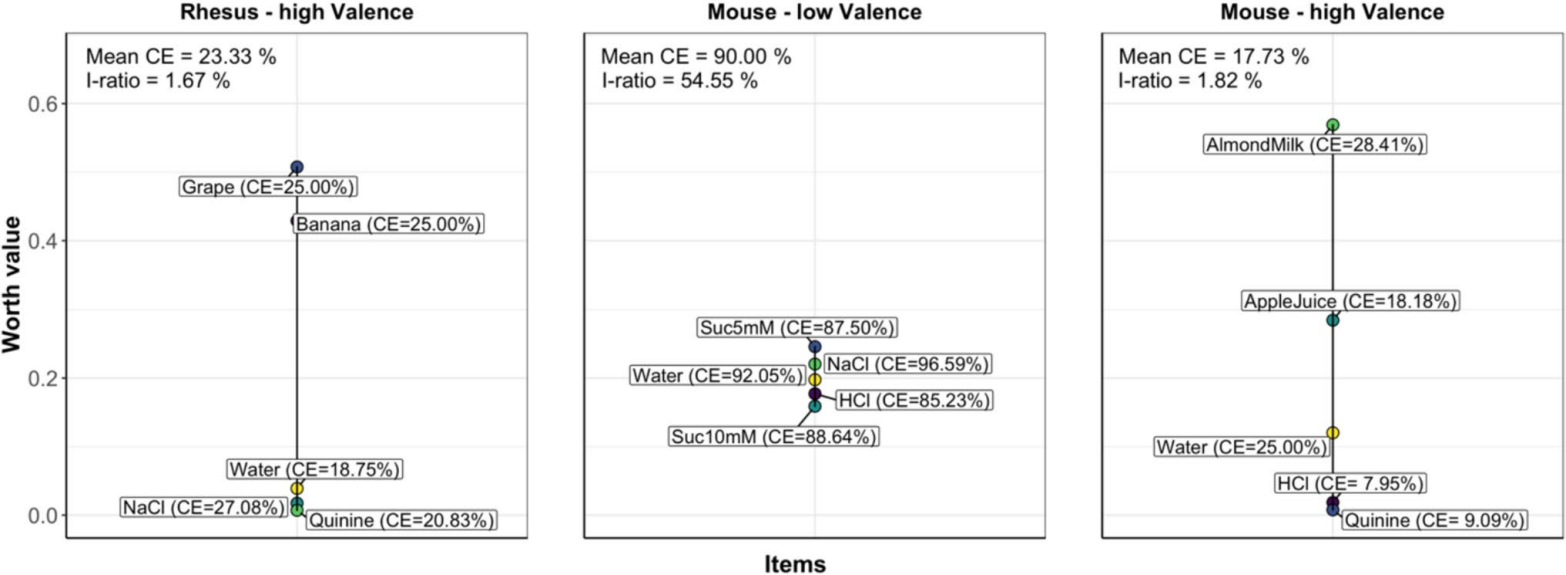
Preference ranking of liquids presented to mice and rhesus macaques with the preference threshold set to 65%. The consensus error (CE; higher values indicate more disagreement between subjects) and the intransitivity ratio (I-ratio; higher values indicate more intransitive choices) are given as quality measures. *Note 1:* The percent transitivity can be derived as 100 – (I-ratio). *Note 2:* This figure does not include results for the human data set, as choices were binary answers (i.e., 50% threshold level)

Increasing the threshold of preference to 65% (Fig. 2), the ranking was comparable to the ranking at the lower threshold of 50% (Fig. 1), except for a slight change in the position of the previously equally ranked banana and grape liquids. The consensus error increased marginally to 23.33%, and an intransitivity ratio of 1.67% indicated only a few intransitive individual choices.

### Mice

The mice were offered two different series of liquids that were prospectively low (first set) or high (second set) in the valence range. Among the liquids with a high valence range, almond milk was the most preferred, followed by apple juice (Fig. 1). Bitter- (quinine) and sour-tasting (HCl) liquids were avoided. Overall, liquids of the high valence range were ranked with low discrepancies between individuals, indicated by an overall consensus error of 16.36%. There were no intransitive choices made. Conversely, in the low-valence-range liquids, the overall consensus error reached 85.46%, indicating considerable disagreement regarding the ranking of the liquids. Counter-intuitively, the 5 mM sucrose solution was ranked higher than the sweeter 10 mM sucrose solution. Interestingly, the intransitivity ratio was 31.82%, indicating that many individual decisions were intransitive (Fig. 1).

When increasing the preference threshold to 65% (Fig. 2), the liquids testes within the high-valence-range block were ranked in the same order as with the 50% threshold. The consensus error increased slightly to 17.73%, and an intransitivity ratio of 1.82% indicated a few intransitive individual choices when the threshold was higher. When increasing the threshold to 65%, no clear ranking for the liquids in the low-valence-range test block could be revealed, indicated by a large overall consensus error of 90% and an intransitivity ratio of 54.55%.

### Simulations

Tables 1 and 2 show the simulated results based on the data from mice for all single liquids versus any combination of elements from the ground truth (column “tested against”). Table 1 represents the high-valence data set, and Table 2 represents the low-valence data set. The tables show the best-achieved result for any of the 200 simulations. Results are sorted by lowest achieved intransitivity ratio followed by lowest consensus error when no prediction or cutoff regarding transitivity of the data is made (i.e., uninformed simulation). The reason behind this sorting is to find the option with the best possible quality measures (intransitivity ratio, consensus error) in a simulation, contrary to using the mean value. The derived rank on the worth-value scale is assumed to be the putative position of the simulated liquid, which was compared to the previously known “true” position, resulting in the provided “number of true positives” in Tables 1 and 2. The fraction of true positives is shown in the tables as the “frequency of true positives.''As a general trend, the greater the number of liquids from the ground truth (column “tested against”) that were included in the simulation, the more often the simulated liquid is ranked to the assumed true position. Our example simulation indicates that adding options to the ground truth (“tested against”) not only improves the overall intransitivity ratio and consensus error but also increases the frequency in which the simulated position of the option replicates its true position (**“**frequency of true positives**”** converging towards 1). In comparison to the low-valence-range data (Table 2), the high-valence-range data (Table 1) shows lower values in the intransitivity ratio (less intransitive) as well as lower values of consensus error (more agreement between subjects).

**Table 1:** Results of the simulation for mice data with high valence range (200 runs, uninformed simulation). The table shows the best-achieved result for any of the 200 simulations. Results are sorted by lowest achieved intransitivity ratio followed by lowest consensus error. The last line in each item section depicts the full set of binary combinations and reflects the true values for item position, matching worth value and CE in Fig. 1, mouse high-valence data

Item	Simulated position	Worth value	I-ratio	CE (%)	Frequency of true positives	No. of true positives	Tested against
Almond milk	1	0.51	0.05	31.82	0.01	2	Apple juice
	3	0.12	0.09	59.09	0.01	1	Water
	2	0.34	0.04	45.45	0.02	4	HCl
	2	0.29	0.01	40.91	0.02	4	Quinine
	1	0.44	0.04	40.91	0.01	2	Apple juice, water
	1	0.49	0.03	31.82	0.24	47	Apple juice, HCl
	2	0.19	0.02	27.27	0.05	10	Water, HCl
	1	0.47	0.03	36.36	0.31	61	Apple juice, quinine
	2	0.24	0.03	40.91	0.02	3	Water, quinine
	3	0.19	0.00	31.82	0.24	47	HCl, quinine
	1	0.49	0.03	31.82	0.56	112	Apple juice, water, HCl
	1	0.49	0.02	31.82	0.55	110	Apple juice, water, quinine
	1	0.61	0.00	13.64	0.99	198	Apple juice, HCl, quinine
	1	0.56	0.00	22.73	0.26	52	Water, HCl, quinine
	1	0.52	0.00	27.27	1.00	200	Apple juice, water, HCl, quinine
Apple juice	1	0.31	0.05	31.82	0.00	0	Almond milk
	2	0.30	0.05	54.55	0.29	58	Water
	3	0.17	0.02	47.73	0.13	26	HCl
	3	0.26	0.02	45.45	0.10	20	Quinine
	2	0.26	0.05	27.27	0.03	5	Almond milk, water
	3	0.21	0.03	34.09	0.01	1	Almond milk, HCl
	2	0.26	0.02	15.91	0.81	161	Water, HCl
	3	0.16	0.01	45.45	0.03	5	Almond milk, quinine
	1	0.42	0.01	40.91	0.72	143	Water, quinine
	2	0.34	0.00	25.00	0.33	66	HCl, quinine
	2	0.30	0.01	20.45	0.75	150	Almond milk, water, HCl
	2	0.29	0.01	22.73	0.55	110	Almond milk, water, quinine
	2	0.27	0.00	25.00	0.10	19	Almond milk, HCl, quinine
	2	0.30	0.00	20.45	0.26	52	Water, HCl, quinine
	2	0.30	0.00	20.45	1.00	200	Almond milk, water, HCl, quinine
Water	3	0.16	0.02	59.09	0.80	160	Almond milk
	3	0.10	0.03	45.45	0.575	115	Apple juice
	3	0.18	0.02	45.45	0.565	113	HCl
	2	0.28	0.03	40.91	0.54	108	Quinine
	3	0.25	0.00	36.36	0.83	166	Almond milk, apple juice
	3	0.17	0.03	40.91	0.72	143	Almond milk, HCl
	3	0.08	0.00	13.64	1.00	200	Apple juice, HCl
	3	0.17	0.00	50.00	0.67	134	Almond milk, quinine
	3	0.10	0.01	27.27	0.93	185	Apple juice, quinine
	3	0.20	0.00	31.82	0.08	16	HCl, quinine
	3	0.14	0.02	27.27	1.00	200	Almond milk, apple juice, HCl
	3	0.14	0.00	27.27	0.85	170	Almond milk, apple juice, quinine
	3	0.15	0.00	22.73	0.11	22	Almond milk, HCl, quinine
	3	0.11	0.00	13.64	0.99	198	Apple juice, HCl, quinine
	3	0.15	0.00	22.73	1.00	200	Almond milk, apple juice, HCl, quinine
HCl	4	0.08	0.01	40.91	0.97	193	Almond milk
	4	0.08	0.04	61.36	0.99	198	Apple juice
	4	0.06	0.06	36.36	0.98	195	Water
	2	0.26	0.03	65.91	0.09	18	Quinine
	4	0.06	0.00	29.55	0.86	171	Almond milk, apple juice
	5	0.04	0.01	27.27	0.80	159	Almond milk, water
	4	0.06	0.02	11.36	0.88	176	Apple juice, water
	4	0.05	0.01	34.09	0.44	88	Almond milk, quinine
	4	0.05	0.00	31.82	0.55	110	Apple juice, quinine
	4	0.05	0.05	25.00	0.96	192	Water, quinine
	5	0.03	0.00	6.82	0.56	112	Almond milk, apple juice, water
	4	0.02	0.00	9.09	0.88	175	Almond milk, apple juice, quinine
	4	0.02	0.00	6.82	1.00	200	Almond milk, water, quinine
	4	0.03	0.03	13.64	1.00	200	Apple juice, water, quinine
	4	0.02	0.00	4.55	1.00	200	Almond milk, apple juice, water, quinine
Quinine	4	0.04	0.02	50.00	0.01	1	Almond milk
	3	0.11	0.04	54.55	0.00	0	Apple juice
	4	0.04	0.12	56.82	0.02	3	HCl
	4	0.03	0.07	50.00	0.01	2	Water
	4	0.03	0.00	31.82	0.05	10	Almond milk, apple juice
	5	0.02	0.04	29.55	0.40	80	Almond milk, HCl
	5	0.01	0.03	11.36	0.24	47	Apple juice, HCl
	4	0.01	0.01	31.82	0.11	22	Almond milk, water
	4	0.01	0.01	31.82	0.075	15	Apple juice, water
	5	0.02	0.10	25.00	0.39	77	HCl, water
	5	0.01	0.01	11.36	1.00	199	Almond milk, apple juice, HCl
	4	0.02	0.00	9.09	0.45	90	Almond milk, apple juice, water
	5	0.01	0.01	6.82	1.00	200	Almond milk, HCl, water
	5	0.01	0.04	15.91	0.99	198	Apple juice, HCl, water
	5	0.01	0.00	6.82	1.00	200	Almond milk, apple juice, HCl, water

*CE* = consensus error, *I-ratio* = intransitivity ratio

**Table 2:** Results of the simulation of mice data with a low valence range (200 runs, uninformed simulation). The table shows the best-achieved result for any of the 200 simulations. Results are sorted by lowest achieved intransitivity ratio followed by lowest consensus error. The last line in each item section depicts the full set of binary combinations and reflects the true values for item position, matching worth value, and CE in Fig. 1, mouse low-valence data

Item	Simulated position	Worth value	I-ratio	CE (%)	Frequency of true positives	No. of true positives	Tested against
Suc5mM	4	0.20	0.23	81.82	0.47	93	Suc10mM
	1	0.26	0.24	73.86	0.19	37	Water
	5	0.12	0.22	77.27	0.28	55	NaCl
	1	0.44	0.24	60.23	0.45	89	HCl
	1	0.31	0.25	78.41	0.41	81	Suc10mM, water
	2	0.24	0.23	68.18	0.58	115	Suc10mM, NaCl
	1	0.43	0.22	60.23	0.28	56	Water, NaCl
	2	0.24	0.26	69.32	0.77	154	Suc10mM, HCl
	1	0.47	0.25	56.82	0.38	75	Water, HCl
	1	0.35	0.25	73.86	0.48	95	NaCl, HCl
	2	0.23	0.25	87.50	0.56	112	Suc10mM, water, NaCl
	1	0.37	0.28	70.45	0.85	170	Suc10mM, water, HCl
	2	0.24	0.28	69.32	0.89	178	Suc10mM, NaCl, HCl
	1	0.34	0.26	75.00	0.50	100	Water, NaCl, HCl
	1	0.31	0.31	79.55	1.00	200	Suc10mM, water, NaCl, HCl
Suc10mM	2	0.23	0.15	72.73	0.28	55	Suc5mM
	3	0.18	0.18	84.09	0.22	43	Water
	5	0.12	0.19	79.55	0.15	30	NaCl
	2	0.27	0.19	75.00	0.24	48	HCl
	5	0.12	0.19	79.55	0.21	42	Suc5mM, water
	2	0.2	0.24	84.09	0.27	53	Suc5mM, NaCl
	5	0.06	0.23	54.55	0.23	46	Water, NaCl
	1	0.32	0.23	61.36	0.40	79	Suc5mM, HCl
	1	0.41	0.23	63.64	0.33	65	Water, HCl
	3	0.24	0.23	77.27	0.27	53	NaCl, HCl
	5	0.12	0.25	81.82	0.12	23	Suc5mM, water, NaCl
	2	0.28	0.24	68.18	0.73	146	Suc5mM, water, HCl
	3	0.19	0.26	72.73	0.44	88	Suc5mM, NaCl, HCl
	3	0.20	0.27	75.00	0.37	74	Water, NaCl, HCl
	2	0.22	0.31	79.55	1.00	200	Suc5mM, water, NaCl, HCl
Water	4	0.14	0.23	73.86	0.25	49	Suc5mM
	5	0.11	0.25	79.55	0.22	43	Suc10mM
	4	0.14	0.20	87.50	0.15	30	NaCl
	2	0.28	0.24	78.41	0.21	42	HCl
	5	0.12	0.24	80.68	0.31	61	Suc5mM, Suc10mM
	4	0.17	0.23	93.18	0.22	43	Suc5mM, NaCl
	1	0.34	0.26	71.59	0.33	65	Suc10mM, NaCl
	4	0.12	0.28	79.55	0.27	54	Suc5mM, HCl
	1	0.30	0.28	80.68	0.21	41	Suc10mM, HCl
	3	0.20	0.24	93.18	0.19	38	NaCl, HCl
	5	0.13	0.24	81.82	0.31	62	Suc5mM, Suc10mM, NaCl
	2	0.23	0.29	90.91	0.27	53	Suc5mM, Suc10mM, HCl
	3	0.19	0.28	94.32	0.24	47	Suc5mM, NaCl, HCl
	3	0.21	0.29	95.45	0.48	96	Suc10mM, NaCl, HCl
	3	0.20	0.33	96.59	1.00	200	Suc5mM, Suc10mM, NaCl, HCl
NaCl	4	0.18	0.19	86.36	0.43	85	Suc5mM
	4	0.14	0.23	88.64	0.46	91	Suc10mM
	1	0.27	0.17	82.95	0.44	88	Water
	5	0.07	0.22	65.91	0.48	96	HCl
	2	0.22	0.24	88.64	0.55	109	Suc5mM, Suc10mM
	2	0.22	0.15	87.50	0.50	99	Suc5mM, water
	1	0.37	0.21	67.05	0.56	112	Suc10mM, water
	4	0.14	0.23	88.64	0.45	89	Suc5mM, HCl
	3	0.19	0.27	95.45	0.56	111	Suc10mM, HCl
	5	0.09	0.19	71.59	0.51	102	Water, HCl
	2	0.25	0.22	80.68	0.71	141	Suc5mM, Suc10mM, water
	4	0.12	0.31	81.82	0.45	90	Suc5mM, Suc10mM, HCl
	4	0.15	0.22	89.77	0.75	150	Suc5mM, water, HCl
	4	0.12	0.27	82.95	0.50	100	Suc10mM, water, HCl
	4	0.18	0.30	96.59	1.00	200	Suc5mM, Suc10mM, water, HCl
HCl	3	0.17	0.15	82.95	0.49	98	Suc5mM
	5	0.08	0.15	61.36	0.73	145	Suc10mM
	1	0.27	0.16	82.95	0.36	71	Water
	1	0.31	0.16	79.55	0.29	57	NaCl
	5	0.08	0.18	67.05	0.88	175	Suc5mM, Suc10mM
	5	0.11	0.18	79.55	0.56	111	Suc5mM, water
	5	0.04	0.16	39.77	0.76	151	Suc10mM, water
	2	0.27	0.19	62.50	0.51	101	Suc5mM, NaCl
	5	0.14	0.19	72.73	0.81	161	Suc10mM, NaCl
	4	0.19	0.21	94.32	0.37	74	Water, NaCl
	5	0.06	0.22	59.09	0.97	194	Suc5mM, Suc10mM, water
	5	0.12	0.22	69.32	0.98	195	Suc5mM, Suc10mM, NaCl
	5	0.10	0.25	77.27	0.75	150	Suc5mM, water, NaCl
	5	0.10	0.25	73.86	0.96	192	Suc10mM, water, NaCl
	5	0.10	0.31	75.00	1.00	200	Suc5mM, Suc10mM, water, NaCl

*CE *= consensus error, *I*-ratio = intransitivity ratio

**Fig. 3:**
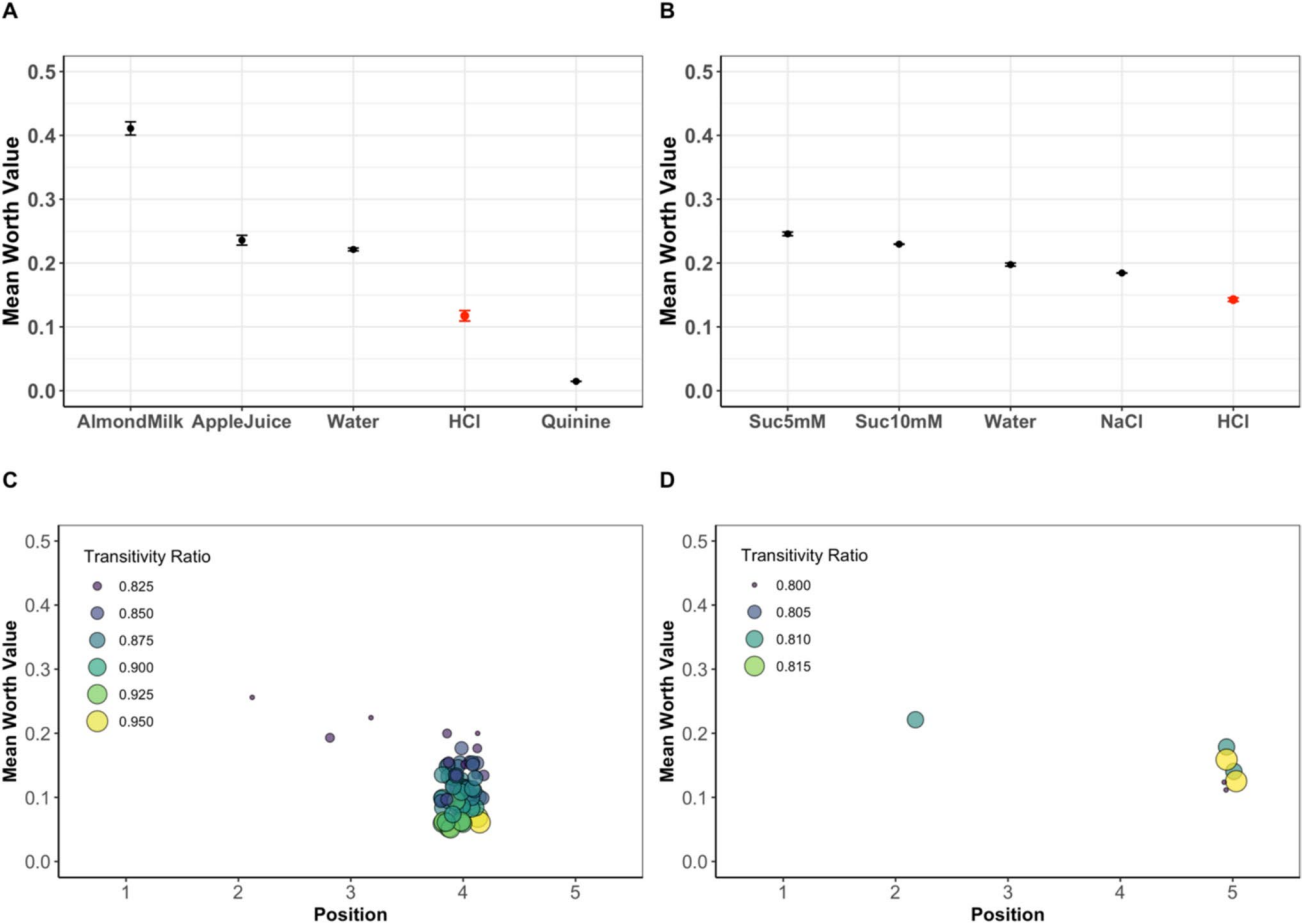
Simulation results for the HCl item in two different sets of mouse data. **A** In the high-valence data, HCl was tested against the items water and quinine in an uninformed simulation, resulting in position no. 4 after 71 randomization runs. The simulated HCl position shows no overlap in 95% confidence intervals with any other item. **B** In the low-valence data, HCl was tested against the items m10MSac and NaCl in an uninformed simulation, resulting in position no. 5 after 496 randomization runs. The simulated HCl position shows no overlap in 95% confidence intervals with any other item. **C** In the informed simulation of the high-valence data, most simulated HCl positions are found in position no. 4 (96%), together with the highest achieved intransitivity ratio after 71 randomization runs (minimum intransitivity ratio = 0.2). In both low- and high-valence cases, the simulated HCl item was placed in the same position as found in the full-worth model. **D** In the informed simulation of the low-valence data, the majority of simulated HCl positions are found in position no. 5 (86%), together with the highest achieved intransitivity ratio during the 496 randomization runs (minimum intransitivity ratio = 0.2)

As an example, we simulated the position of HCl in both the high-valence (simulated against water and quinine) and low-valence (simulated against Suc10 mM and NaCl) data (see Fig. 3). Here, first, we used an uninformed simulation method, i.e., no restrictions in terms of allowed intransitivity (Fig. 3A and B). The simulations resulted in the correct rank of HCl (according to the ground truth) in both the high-valence data (HCl = rank 4) and the low-valence data (HCl = rank 5) with high precision. The results were confirmed using informed simulations, i.e., with a preset ratio of tolerated intransitivity of 0.2 (Fig. 3C and D). We deliberately used a cutoff value of 0.3 as the maximum allowed intransitivity ratio and included the simulated intransitivity ratios as an additional quality measure of the simulations. Here, most simulations yielded the correct positions, and wrong positions were only simulated with relatively high intransitivity ratios (low-valence data: intransitivity ratios > 0.25, high-valence data: intransitivity ratios > 0.175).

## Discussion

We used multiple binary comparisons to rank different options exemplified by data from humans, rhesus macaques, and mice. Rankings were derived using worth values to determine the preference strength (Grand et al., 2017; Hatzinger & Dittrich, 2012). An additional benefit of using worth values is the added information of an option’s scaled position within a ranking. To evaluate the quality of such scaled ratings, we introduced two new quality measures (consensus error and intransitivity ratio). Using simulations, we investigated the feasibility of introducing new options into existing rankings without the need to perform all possible binary choice tests. By applying our quality measures, researchers can gauge the performance and positioning of these newly integrated options and effectively minimize the need for extensive testing.

In all three species, binary choice tests were conducted, which led to rankings derived from calculated worth values. In humans, the ratings of different pictures in our studies were scaled along the predicted valence scores that were published with the OASIS data (Kurdi et al., 2017), i.e., pictures with higher valence scores in the OASIS study resulted in higher worth values in our studies. This pattern of agreement was true for image sets with both high and low valence ranges.

While the valence data were available in humans, we had to assume differences concerning the valences in the tested liquids presented to rhesus macaques and mice. Not surprisingly, in rhesus macaques, the sweet-tasting juices (grape and banana) were most preferred by all individuals, while salty and bitter-tasting liquids (NaCl and quinine, respectively) were least preferred. Water was also rated low, possibly indicating the unique nature of the rewarding fruity liquids. Likewise, sweet-tasting liquids (sucrose, almond milk, and apple juice) were preferred over water and salty- (NaCl), sour- (HCl), and bitter-tasting (quinine) fluids in mice. This general acceptance for sweet and avoidance for bitter tastes is also known for most humans and reflect the basic ecological facts that in nature, sweetness signals calories, while bitterness predicts toxicity (e.g., Rozin, 2009; Ventura & Worobey, 2013), although genetic differences in sensitivity to taste bitterness exists in humans (e.g., in respect to the prevalence of malaria; Krebs, 2009). It is worth noting that while in our experimental data the preferences can be affected by different internal and external factors (e.g., genetic, familiarity, culture, social, individual motivation; Krebs, 2009; Rozin, 2009), the method of combining binary choice tests into a scaled ranking is independent of the reasoning behind the preferences.

While the derived worth values resulted in clear rankings, the separability of the options within the ratings differed considerably. In rhesus macaques, for instance, the two juices (grape and banana) were differentiable from all others, while no clear preferences were found between them. The range of valence differences between the individual options played an important role here. Overall, the more similar the rated options were to each other, the more difficult it was to reveal a conclusive preference ranking. This can be exemplified by imagining a person preferring sugared coffee over black coffee. While there is, for example, no measurable difference between 2.99 g and 3 g of sugar, there would be a clear preference for 3 g over 0 g.

To investigate the effect of differences in valence, we worked with data of different valence bandwidths in humans and mice. We tested the quality of the ratings by calculating the consensus error and the intransitivity ratio. Options closer in valence (human: valence known from OASIS data; mice: presumed differences in valence) resulted in ratings closer in worth values, compared to options ranging on a broader valence scale. Our quality measures accurately reflected these differences in rankings between the different valence ranges, i.e., larger consensus error and intransitivity ratio in low-valence than in high-valence data.

Many nonhuman animal studies use multiple binary comparisons to obtain rankings where only the relative position of the options is known. For example, the study by Schroeder et al. (2014) established a hierarchy of preferences using the percentage of tank occupancy time during preference tests for different options of enrichment chosen by zebrafish (*Danio rerio*). Using time spent in differently enriched boxes or in proximity to containers loaded with different scents was also used as an indication of preference in rats (*Rattus norvegicus*, Manser et al., 1998) and giraffes (*Giraffa camelopardalis rothschildi*, Fay & Miller, 2015), respectively. Although they provide a valuable indication of the order in which options are preferred, these studies were inconclusive concerning how much more or less a particular option was preferred over another.

In addition to a mere ranking, binary choice tests can be used to calculate the scaled positions of different options so that the relationships within rankings are known. This calculation has been done, for example, in studies evaluating food preferences in golden lion tamarins (*Leontopithecus rosalia*, Benz et al., 1992) and great apes (gorillas, *Gorilla gorilla gorilla;* chimpanzees, *Pan troglodytes:* Remis, 2002). Golden lion tamarins preferred two food items (raisin and mealworm), while all other food items were chosen less frequently. The difference in preferences between raisins and mealworms was small, while the difference to the next food type in the scaling (egg) was considerably larger. Both publications (Benz et al., 1992; Remis, 2002) used *z*-scores to scale different food types. However, the *z*-score approach assumes a Gaussian distribution of preferences, which may not reflect the actual distribution of events. This method should therefore be applied with caution. In our view, rare events and skewed choices are better represented by probabilistic models such as the log-linear Bradley–Terry model (Bradley & Terry, 1952). Thus, the efficient ranking and scaling of the resulting worth values offer better informational insights than *z*-scores. Combined with our quality measures, we consider the use of worth values superior to conventionally applied scaled ranking methods (*z*-score) and an important step forward when evaluating preferences. For a method comparison, we provide *z*-scores after Remis (2002) in our supplementary material, Fig. S2.

Yet another feature that improves the interpretation of preference tests that we provide in our “simsalRbim” package is the possibility to choose different preference thresholds or cutoffs for preferences. For instance, defining an option as preferred only if selected, e.g., in over 65% of cases (rather than 50%) can provide a more apparent distinction and reduce the ambiguity of results. Laska, for instance, investigated gustatory preferences for different concentrations of sugars and acids, respectively, in nonhuman primate species (pigtail macaques, *Macaca nemestrina:* Laska, 2000; squirrel monkeys, *Saimiri sciureus:* Laska, 1994, 1996, 1997, 1999; spider monkey, *Ateles geoffroyi*: Laska et al., 1996, 1998; baboons, *Papio hamadryas*: Laska et al., 1999) and European rabbits (*Oryctolagus cuniculus*, Laska, 2002), applying 67.7% of the total amount consumed as the threshold for preference. The author noted that this rather conservative criterion was used to “avoid misinterpretation of data due to too-liberal criterion” (Laska et al., 2001).

We can show the effect of such preference thresholds using the quality measures we introduced. In the human data, all choices were precisely set to the threshold level of 50%, i.e., participants were given the choice between one picture and the other. Therefore, in our data sets, the effect of changes due to different threshold levels could only be demonstrated in mice and rhesus macaques. While in rhesus macaques grape was indistinguishable from banana at the 50% threshold, it was ranked in first position at the 65% threshold level. On the contrary, no differences in rankings were found in mice in the high-valence data, regardless of the set threshold level. However, several options'rank positions differed depending on the set threshold level in the low-valence mice data. In all instances, our two quality measures indicated a lower quality of the rankings with the 65% threshold, i.e., an increase in mean consensus error and intransitivity ratio. This loss in quality indicates that data with less transitive and discordant answers, as is the case for our low-valence data, and higher thresholds for the preference cutoff do not lead to more conclusive results. In our example, applying a higher preference threshold even resulted in an unintuitive change in ranking, i.e., one of the sucrose liquids was ranked least preferred. In the high-valence data sets, the effect of changing the threshold to 65% was only marginal. A primary disadvantage of setting a threshold other than 50% is the increased chance of generating ties. If only decisions larger than the set threshold are considered a true preference, the remaining choices below the threshold must be considered indecisive. Such incomplete preference information requires different strategies to achieve meaningful results, i.e., collecting data without ties and simulating uncertainties.

A unique feature of our package is the possibility for simulated positions of options with only incomplete preference information available. The information gained from these simulations will allow future rankings and scaling to be created at reasonable precision without necessarily having to perform all individual comparisons. Our simulation-based approach with empirical data proposes a workflow for generating rankings from incomplete binary comparisons. We suggest two types of simulations differing with respect to assumptions made for intransitivity. The uninformed simulation does not require assumptions about whether the derived order is based on intransitive decisions. In the informed simulation, the desired grade of intransitivity can be included to derive rankings that reflect a certain degree of intransitive choices. Using the mouse low- and high-valence data, we used both simulation types to determine the position of the sour-tasting liquid (HCl). To achieve this, HCl was eliminated from both sets and reentered for the simulations. In both simulations, the position of HCl was correctly identified (Fig. 3).

The uninformed simulation is a twofold process. In the first step, a cutoff value is determined statistically to find a heuristic threshold for the number of required simulation runs to obtain a stable position of the queried option within the whole set of options. The queried option’s position is compared to the other options using the worth value calculation at each randomization step. At each step of the cutoff determination, the position errors are calculated from the multiple randomizations using a linear model, i.e., yielding an adjusted *p*-value. Therefore, increasing the number of simulations constricts the limited error space, resulting in smaller confidence intervals. The optimal position for an option is reached when the 95% confidence interval of the queried option during the cutoff calculation is smaller than the significance threshold of *p* ≤ 0.05. However, when such a threshold cannot be obtained in a reasonable number of runs (depending on available computation power), the queried option’s position is ambiguous. The second step involves the implementation of the cutoff value to simulate the new option’s position, representing the computationally most efficient solution for the new option’s position. For orientation, our package (“simsalRbim”) offers functionalities (“*bimUninformed”*) to determine the cutoff value heuristically, i.e., the number of required randomization runs for complete option separation. These cutoffs can be used as seeding points in the analysis. The user can also use arbitrary cutoffs. However, smaller than optimal values will result in less precise rankings, while larger than optimal cutoff values will lead to increased computation time without adding further value. In Fig. 3, for example, the simulated options’ worth values are shown with 95% confidence intervals, resulting from the prior determined cutoff value. In the case of Fig. 3A, the high-valence data required a cutoff of 71 simulation runs, while the low-valence data (Fig. 3B) needed 493 runs to separate all options. In cases of highly overlapping data, complete separation of options might require too much computation time or would not be possible at all. In such cases, one can try to separate the options in question heuristically with a reasonable number of iterations by trial and error until there is no or minimal overlap of the confidence interval with any other options. For example, this approach could have found the same positions of the option in question (HCl) simulated in Fig. 3 after only 20 iterations.

While an uninformed simulation can give a reasonable estimate of the rank position of a newly introduced option, these results might reflect a considerable number of simulated intransitive choices. If the data in question allow us to reasonably estimate the tolerable intransitivity, this can be included by using the informed simulation. In Fig. 3C and D, such informed simulations were performed, requiring a maximum intransitivity ratio of 0.2 (i.e., less than 20% of option choices are intransitive). In the current example, uninformed and informed simulations yield similar results regarding the positions of the simulated option. The additional value of using the informed simulation is the knowledge that such ratings can be achieved even when excluding excessive intransitivity. Note that performing an informed simulation has a higher computational demand. Thus, starting with the uninformed simulation might be a good idea.

Preference tests are also a straightforward means of addressing animal welfare-related questions, for example, testing preferences for different kinds of food (Yamada, 2017), husbandry conditions like bedding material (Patterson-Kane et al., 2001; Ras et al., 2002), cage size (Dawkins, 1977) and cage enrichment (Hobbiesiefken et al., 2023; Lewejohann & Sachser, 2000), cognitive tasks (Calapai et al., 2023), conspecifics (Pfefferle et al., 2014), or experimental or caretaking procedures (Fitchett et al., 2006; Millot et al., 2014). One drawback of such tests is that knowledge about a preference for one option over another does not allow direct inference on distress or welfare an animal might or might not experience (discussed in Dawkins, 1977; Kirkden & Pajor, 2006). For example, luxury items might be preferred at low costs, but less of the luxury item would be consumed if the price is increased (“elastic demand”). On the contrary, the demand for necessities will be less affected by an increasing price because it is a constant requirement (“inelastic demand”; discussed in Fraser & Matthews, 1997; Kahnau et al., 2020, 2023; Kirkden & Pajor, 2006; Stamp Dawkins, 1988). Elaborating on this example further, food is an undeniable necessity, and food deprivation will lead to suffering in the long run. Other options like access to litter (hens, *Gallus gallus domesticus*, Stamp Dawkins, 1983), additional social contact (rats, *Rattus norvegicus,* Patterson-Kane et al., 2002), or additional non-enriched space (mice, *Mus musculus*, Lewejohann & Sachser, 2000) have a more flexible demand. Here, however, interpretations should be made carefully because a flexible demand does not imply per se that this option is unnecessary. It can only be assumed that denying this option causes less suffering than denying options with less flexible demands (discussed in Stamp Dawkins, 1983). In addition, all shown preferences are context-dependent. They can be influenced by previous experience (Dawkins, 1977), current motivational state, e.g., food deprivation (Stamp Dawkins, 1983; Yamada, 2017), or factors that are not instantly obvious (discussed in Fraser & Matthews, 1997).

Overall, preference testing is a window into the animal’s perspective. Based on preference rankings, researchers will be able to choose the most effective reinforcer for an experiment (Gaalema et al., 2011) and reduce aversive response to external factors (Baumans et al., 2002), and in the end might accomplish severity assessment from the animal’s perspective (discussed in Cassidy, 2024; Habedank et al., 2018). Thus, in animal welfare research in particular, preference tests play a pivotal role in better understanding the perspectives of animals.

## Summary

To help tap into an animal’s perspective, we developed and described a method that pushes the boundaries of preference testing. Our approach harnesses multiple binary comparisons to generate scaled rankings of multiple options rather than rankings alone. As ambiguities regularly occur over multiple-binary-preference tests due to missing values or ties, we introduced quality measures (i.e., consensus error and intransitivity ratio) to evaluate the validity of gained scaling. These measures make it possible to improve the ranking and to see where more detailed investigations would be necessary for a more precise evaluation. In addition, our approach allows us to change the threshold for what we call preference, e.g., if we only want to rate an option as preferred if the preference is at least 65%. In such a case, we have observed that inaccuracies (i.e., the number of ties) can increase, especially for options within a small valence range. Our approach addresses this issue through simulations, i.e., by replacing the ties and, thereby, evaluating the influence of the simulated results using the proposed quality measures (consensus error and intransitivity ratio). The desired minimum quality can be freely selected, and thus a satisfactory result can be achieved even with imprecise data. Simulations make it possible to incorporate new options into existing scales without necessarily having to test all individual comparisons, thereby reducing the frequency of animal experiments. Overall, our approach makes it possible to conduct preference tests in animals in a more targeted way, enabling qualified statements about the relative value of certain options.

## Supplementary Information

Below is the link to the electronic supplementary material.

Supplementary file1 (DOCX 9.09 MB)

## Data Availability

The data for this study can be accessed at https://github.com/mytalbot/simsalRbim_data. The R package “simsalRbim” is described in more detail under https://talbotsr.com/simsalRbim/.

## Code availability

The code for the script of the human preference test is available at https://github.com/RefinementReferenceCenter/Prefvalence. The R package “simsalRbim” is available at https://github.com/mytalbot/simsalRbim and is described in more detail under https://talbotsr.com/simsalRbim/.
