# Supplementary material for: Advancing preference testing in humans and animals
Source: Behav Res Methods. 2025 Jun 6;57(7):193. doi: 10.3758/s13428-025-02668-5 (PMC12144046; doi:10.3758/s13428-025-02668-5)
Supplement: Supplementary file 1 — Supplementary file1 (DOCX 9.09 MB) [file 13428_2025_2668_MOESM1_ESM.docx]

Advancing Preference Testing in Humans and Animals

Dana Pfefferle*^1,2^, Steven R. Talbot*^3^, Pia Kahnau^4^, Lauren C. Cassidy^1,5^, Ralf R. Brockhausen^1^, Anne Jaap^6^, Veronika Deikun^7^, Pinar Yurt^1,2,8^, Alexander Gail^1,2,9^, Stefan Treue^1,2,9^ and Lars Lewejohann^4,6^

^1^ Welfare and Cognition Group, Cognitive Neuroscience Laboratory, German Primate Center–Leibniz Institute for Primate Research, Göttingen, Germany

^2^ Leibniz-Science Campus Primate Cognition, German Primate Center & University of Göttingen, Göttingen, Germany

^3^ Institute for Laboratory Animal Science, Hannover Medical School, Hannover, Germany

^4^ German Federal Institute for Risk Assessment (BfR), German Centre for the Protection of Laboratory Animals (Bf3R), Berlin, Germany

^5^ Population and Behavioral Health Services, California National Primate Research Center, University of California, Davis, California, United States

^6^ Institute of Animal Welfare, Animal Behavior and Laboratory Animal Science, Freie Universität Berlin, Berlin, Germany

^7^ Department of Sociobiology/Anthropology, Johann-Friedrich-Blumenbach Institute for Zoology, Georg-August University, Göttingen, Germany

^8^ Georg-August University School of Science, Göttingen, Germany

^9^ Bernstein Center for Computational Neuroscience, Göttingen, Germany

**Table S1**

Image sets from the OASIS database (Kurdi et al., 2017) used for human preference testing

| **High Valence Range** | | **Low Valence Range** | |
| --- | --- | --- | --- |
| Image  (name) | Valence Score | Image  (name) | Valence Score |
| 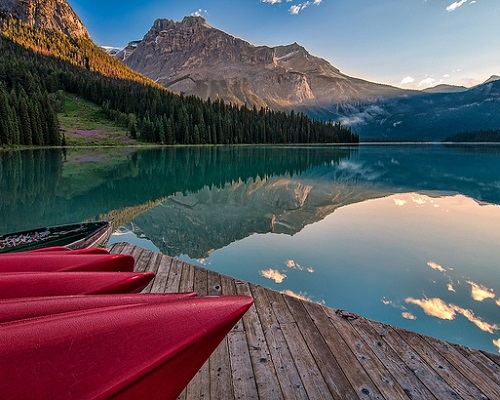  (Lake_9.jpg) | 6.41 | 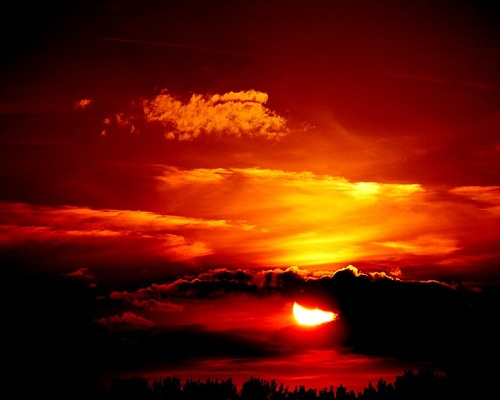  (Sunset_1.jpg) | 6.07 |
| 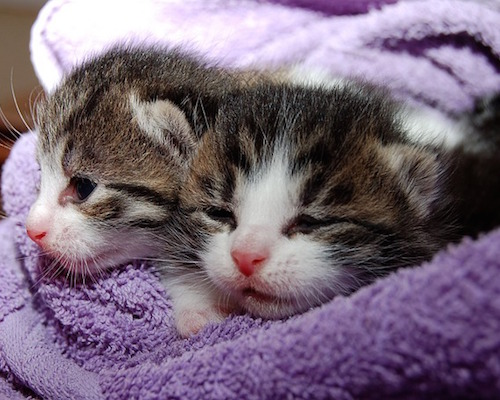  (Cat_5.jpg) | 5.42 | 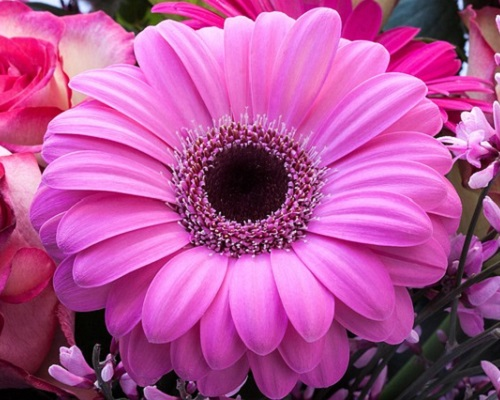  (Flowers_3.jpg) | 5.79 |
| 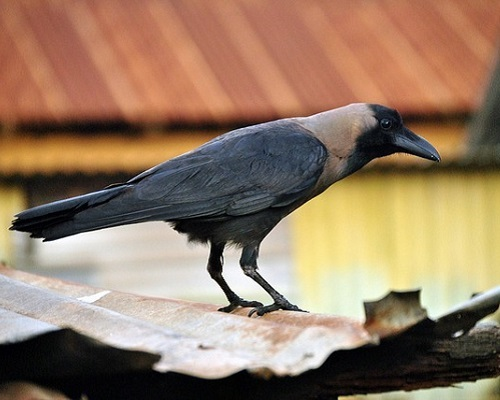  (Crow_1.jpg) | 4.71 | 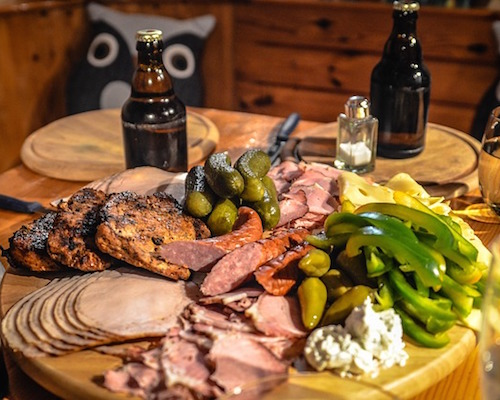  (Food_4.jpg) | 5.61 |
| 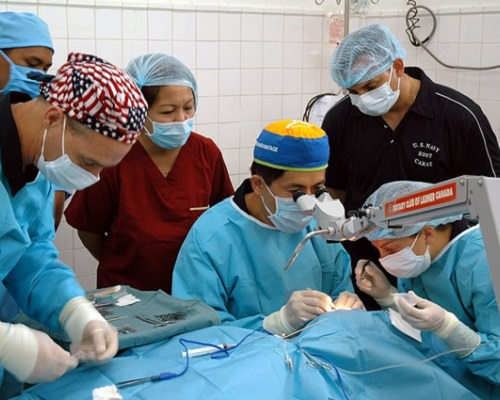(Doctor_4.jpg) | 3.81 | 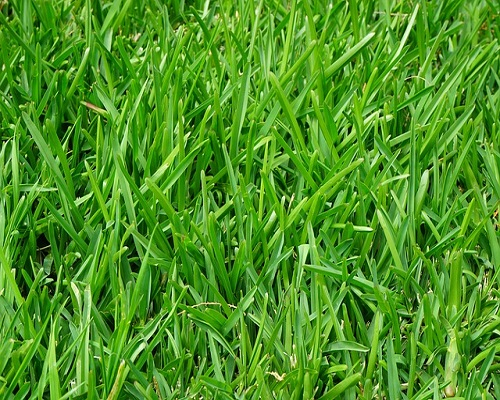(Grass_1.jpg) | 4.96 |
| 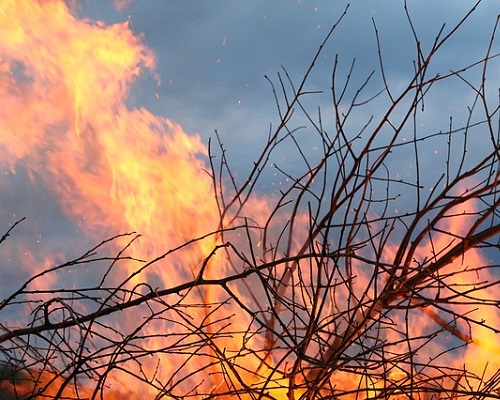(Fire_10.jpg) | 2.47 | 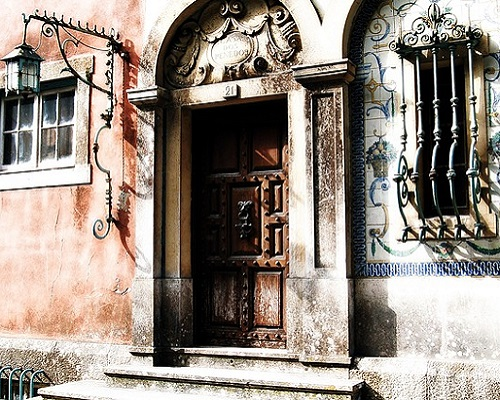(House_1.jpg) | 4.71 |
| 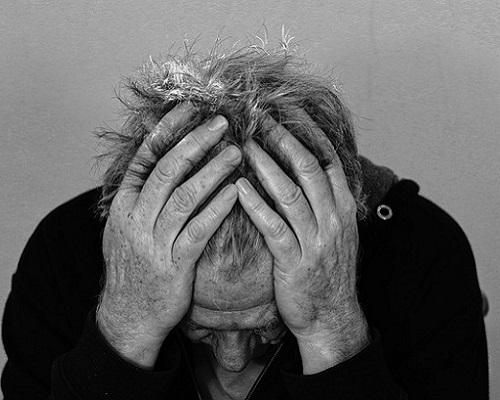(Frustrated_pose_5.jpg) | 2.46 | 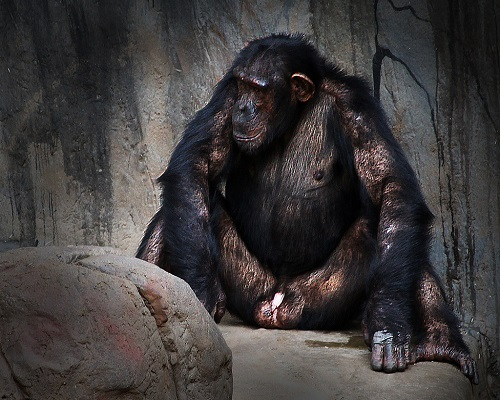(Monkey_2.jpg) | 4.03 |
| 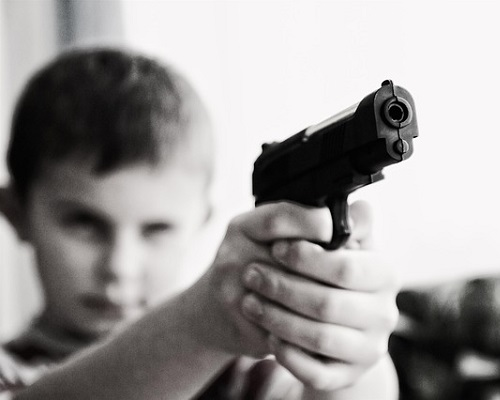(War_1.jpg) | 1.76 | 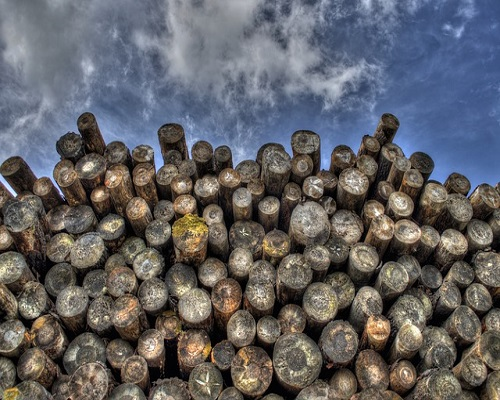(Timber_1.jpg) | 3.83 |

**Table S2**

Overview of the two preference test (low vs. high valence range) run on mice

|  | **Test low valence range**  (1^st^ set of presentation) | **Test high valence range**  (2^nd^ set of presentation) |
| --- | --- | --- |
| Time | April – October 2018 | November - March 2019 |
| Tested liquids | ·10 mM sucrose  ·5 mM sucrose  ·tap water  ·10 mM NaCl  ·10 mM HCl  All liquids were diluted with tap water. | ·1:3 dilution almond milk : tap water  ·1:3 dilution apple juice : tap water  ·tap water  ·10 mM HCl diluted with tap water  ·3 mM quinine hydrochloride dihydrate diluted with tap water |
| Habituation phase | In one corner of the IntelliCage of the test system. | In all four corners of the IntelliCage of the test system. |
| Free water phase | In one corner of the IntelliCage of the test system. | In all four corners of the IntelliCage of the test system. |
| Drinking Sessions | Yes  ·7 – 9 pm  ·0 – 2 am  ·5 – 7 am | No |
| Light/dark circle | Lights on from 8 am to 8 pm | Lights on from 7 am to 7 pm |

**Figure S1**

Home cage based test system. The home cage was connected via an AnimalGate with the IntelliCage where the preference test was realized. The mice received food only in the home cage where also nesting and bedding material was. The IntelliCage has four automated drinking stations (1-4) in which water or test fluids were available.

| 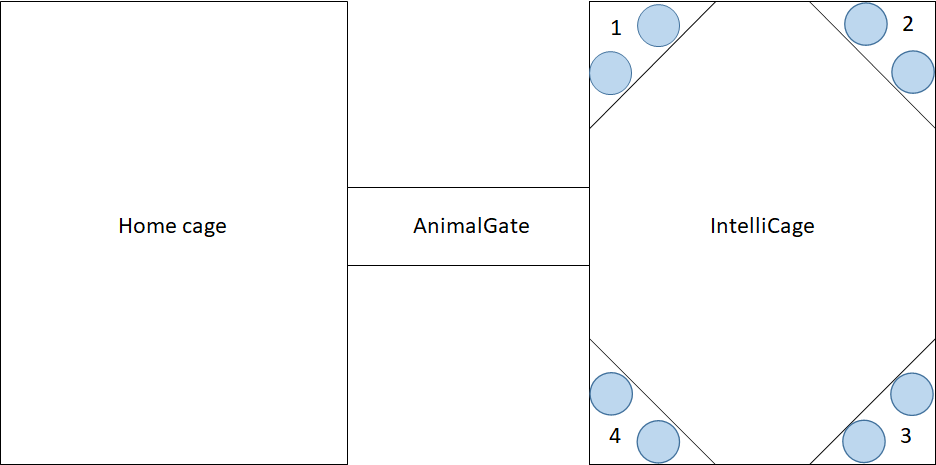 |
| --- |

Comparing scaled rankings derived from worth values calculated following Grand et al. (2017) and Hatzinger & Dittrich (2012) (see main article Fig.1) with result of a z-score calculation after Remis (2002) (SI Fig.1).

Figure S2

Preference ranking of liquids presented to mice, depicting (A) liquids low in valence and (B) high in valence range. Preferences for options were assessed using paired comparisons. For each option, comparisons against all others were generated, and the percentage contribution of the target option was calculated. Options exceeding 50 % were marked as wins. Preference scores were standardized using z-scores and adjusted per subject (see Remis, 2002). Group means were computed to rank the options. Finally, preferences were visualized with a bar plot displaying the adjusted z-scores.

**
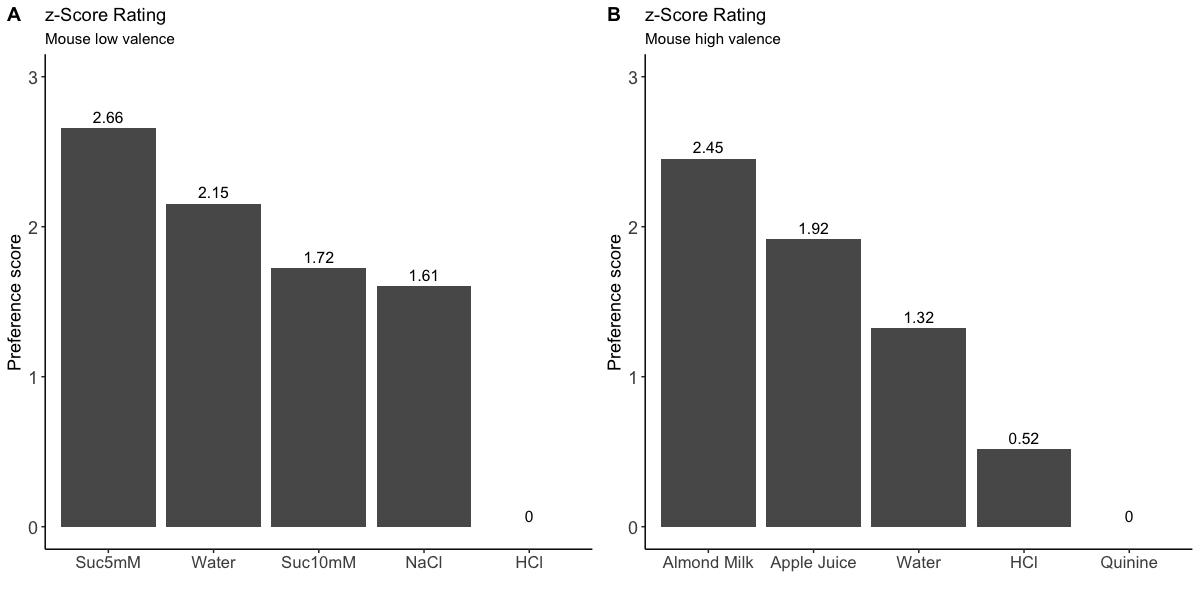
**

While in the large mouse valence dataset the order of options is identical, in the mouse low valence data the positions of water and Suc10mM are switched in comparison to the order found in our worth value calculation. This switch in position is not surprising as in our worth value calculation these options were found to be very close to each other with the according CE values being very high (Water CE: 96.59, Suc10mM CE: 79.55) indicating low consensus among mice regarding these options.

Regarding the scaled information, i.e. how much more likely option A is valued over option B, in the mouse high valance data, our worth values (Fig.1 main article) suggest that Almond Milk (worth value: 0.52) is more preferred over Apple Juice (worth value: 0.3) than Apple Juice over Water (worth value: 0.15), and HCl (worth value: 0.02) is similarly disliked as Quinine (worth value: 0.01) (based on comparing differences in worth values). This is not reflected in the z-score data (Fig. S2), where the options are equally distributed on the z-score scale. These differences might be caused by the z-score approach assuming a Gaussian distribution of data, which does not reflect the actual distribution. We believe rare events and skewed choices are better represented by probabilistic models such as the log-linear Bradley-Terry model (Bradley & Terry, 1952).

# References

Bradley, R. A., & Terry, M. E. (1952). Rank Analysis of Incomplete Block Designs: I. The Method of Paired Comparisons. *Biometrika*, *39*(3/4), 324. https://doi.org/10.2307/2334029

Grand, A., Dittrich, R., & Hatzinger, R. (2017). *Präferenzmodelle in der Praxis: Analyse von Paarvergleichen, Likert Items und Rankings mit R-prefmod*. UTB.

Hatzinger, R., & Dittrich, R. (2012). prefmod: An *R* Package for Modeling Preferences Based on Paired Comparisons, Rankings, or Ratings. *Journal of Statistical Software*, *48*(10). https://doi.org/10.18637/jss.v048.i10

Kurdi, B., Lozano, S., & Banaji, M. R. (2017). Introducing the Open Affective Standardized Image Set (OASIS). *Behavior Research Methods*, *49*(2), 457–470. https://doi.org/10.3758/s13428-016-0715-3

Remis, M. J. (2002). Food Preferences among captive western gorillas (*Gorilla gorilla gorilla*) and Chimpanzees (*Pan troglodytes*). *International Journal of Primatology*, *23*(2), 231–249. https://doi.org/10.1023/A:1013837426426
